# Isolated anomalous left upper pulmonary vein connection in a child: a case report

**DOI:** 10.1097/RC9.0000000000000034

**Published:** 2026-01-02

**Authors:** Alwaleed Al-Dairy, Basheer AlRefai, Bassam Khrma, Ahmad Al-Bitar

**Affiliations:** a Damascus University, Damascus, Syrian Arab Republic; b Faculty of Medicine, Damascus University, Damascus, Syrian Arab Republic

**Keywords:** congenital heart defect, partial anomalous pulmonary venous connection, pulmonary veins, surgical repair

## Abstract

**Introduction:**

Partial anomalous pulmonary venous connection (PAPVC) is a rare congenital heart defect where one or more pulmonary veins fail to connect to the left atrium. Left-sided PAPVC accounts for only 10% of cases, often involving the left upper pulmonary vein (LUPV). While many patients remain asymptomatic, prolonged volume overload in the right heart can lead to symptoms like dyspnea. Non-invasive imaging (TTE/CTA) is critical for diagnosis.

**Case presentation:**

An 11-year-old female presented with dyspnea and recurrent respiratory infections. Imaging revealed isolated LUPV drainage into the innominate vein via an ascending vertical vein, with right heart dilation. Surgical repair involved anastomosing the transected vertical vein to the left atrial appendage under cardiopulmonary bypass. Postoperative recovery was uneventful, with normalized pulmonary venous drainage and symptom resolution at 1-year follow-up.

**Clinical discussion:**

Left-sided PAPVC is rare (10–15% of PAPVC cases) and often asymptomatic, delaying diagnosis. Symptomatic patients require surgery to prevent right heart failure and pulmonary vascular complications. Non-invasive imaging (CTA/MRI) has replaced invasive diagnostics. Surgical success hinges on creating a gradient-free anastomosis; our on-pump approach achieved this, aligning with favorable outcomes reported in the literature.

**Conclusion:**

Early surgical intervention in symptomatic PAPVC prevents irreversible cardiac complications. Accurate imaging and meticulous surgical technique, as demonstrated in this case, are vital for optimal long-term outcomes.

## Introduction

Partial anomalous pulmonary venous connection (PAPVC) represents a sporadic congenital heart defect (CHD) in which one or more of the pulmonary veins (but not all) embryologically fail to connect to the left atrium (LA)^[1]^. The right pulmonary veins are more commonly affected, and only 10% of PAPVC cases involve the left pulmonary veins^[1,2]^. It is more common (nearly twice) for the left-sided PAPVC to affect only the left upper pulmonary vein (LUPV) than the entire left pulmonary veins^[3]^. Associated atrial septal defect (ASD) is usually encountered in PAPVC patients (80–90% of cases); however, an intact atrial septum (IAS) may be the case^[3,4]^. In isolated left-sided PAPVC (with IAS), patients may remain asymptomatic for decades, especially when it involves a single anomalous vein^[2,5]^. Symptoms such as dyspnea, fatigue, exercise intolerance, and palpitations may arise from long-standing PAPVC and subsequent volume overload in the right heart chambers^[6]^. Transthoracic echocardiography (TTE) represents the main diagnostic tool for PAPVC, and computed tomography angiography (CTA) confirms the diagnosis and provides more anatomical details^[6,7]^. Herein, we present an 11-year-old girl who underwent successful surgical repair of an isolated left-sided PAPVC.

**HIGHLIGHTS**

- Early surgical intervention is critical for symptomatic PAPVC patients to prevent long-term complications such as right ventricular failure and irreversible pulmonary vascular disease.
- Accurate diagnosis (via non-invasive imaging like TTE/CTA) and meticulous surgical technique are essential for ensuring successful repair and minimizing postoperative morbidity.

This case report has been reported in line with the SCARE checklist^[8]^.

## Case presentation

An 11-year-old female was referred to the pediatric cardiology clinic at our hospital with a 6-month history of progressive dyspnea on exertion and recurrent lower respiratory tract infections. Her primary care physician, noting the lack of response to bronchodilators and the finding of a widely split S2 on auscultation, initiated the referral to rule out a cardiac etiology. She was not evaluated by a pulmonologist. Her past medical history was unremarkable, with a normal birth history and no known comorbidities. There was no family history of congenital heart disease.

On physical examination, her vital signs were within normal limits: heart rate 88 bpm, blood pressure 102/65 mm Hg, respiratory rate 18 breaths per minute, and oxygen saturation 98% on room air. Auscultation revealed a normal S1 and a widely split S2, but no murmur. The remainder of the examination was unremarkable.

The patient’s symptomatic timeline began 6 months prior to surgery with the onset of dyspnea and an increased frequency of respiratory infections. Due to persistent symptoms, she was referred to pediatric cardiology 1 month prior to surgery. Initial TTE was performed, which raised suspicion for an anomalous pulmonary vein. A definitive diagnosis was confirmed by CTA 3 weeks later. The case was discussed by the multidisciplinary heart team, including pediatric cardiologists and cardiothoracic surgeons, and the decision was made to proceed with elective surgical repair.

Differential diagnoses considered at presentation included asthma, interstitial lung disease, and other forms of congenital heart disease, such as an atrial septal defect.

Laboratory Results: Complete blood count, electrolytes, and renal function tests were all within normal limits. Key results included a hemoglobin of 12.8 g/dl (ref: 11.5–15.5), white blood cell count of 7.1 × 10^3^/µl (ref: 4.5–13.5), and creatinine of 0.6 mg/dl (ref: 0.5–1.0).

TTE revealed significant dilation of the right atrium and right ventricle, suggestive of right heart volume overload. The interventricular septum showed flattening during diastole. Pulmonary venous connections were difficult to visualize completely, but a suspicious flow pattern suggested an anomalous left pulmonary vein. Crucially, an atrial septal defect (ASD) was ruled out, confirming the diagnosis as an isolated PAPVC with an intact atrial septum.

**Figure 1.**
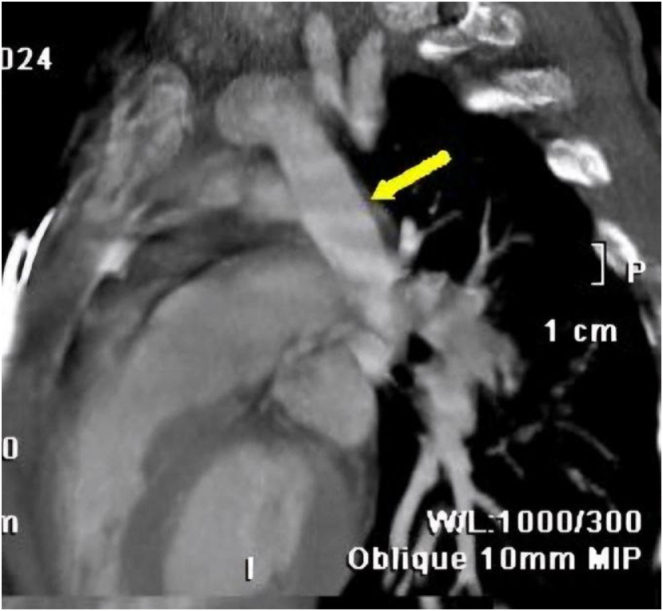
Preoperative CTA image showing the ascending vertical vein (The yellow arrow) draining the left upper pulmonary vein.

**Figure 2.**
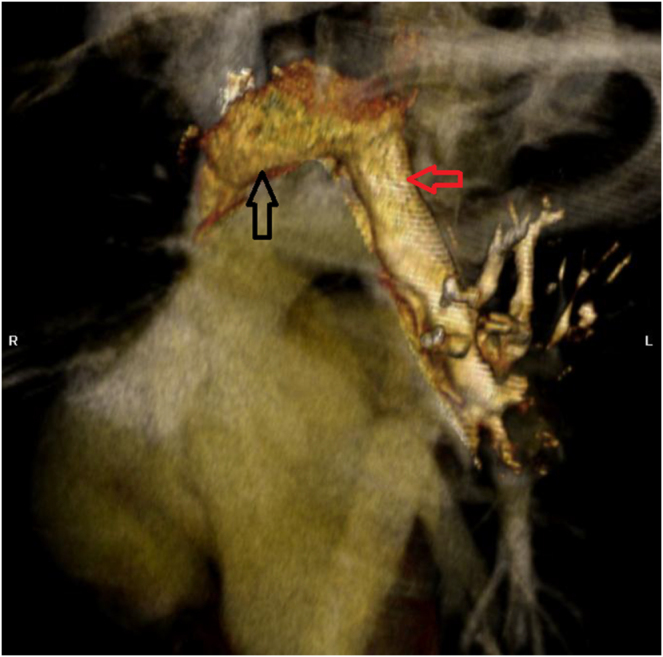
Preoperative CTA image showing the ascending vertical vein (The red Arrow) connecting into the Innominate vein (Black Arrow).

Computed Tomography Angiography (CTA) was performed for definitive diagnosis and surgical planning. It confirmed the isolated anomalous drainage of the LUPV, which connected to the innominate vein via a vertically oriented collateral vein [ascending vertical vein (AVV); Figure 1 and 2]. The other three pulmonary veins were confirmed to have normal drainage into the left atrium.

Given the patient’s symptoms and evidence of right heart volume overload, the decision was made for surgical correction. Alternative management options, such as medical management, were deemed inappropriate due to the risk of progressive right heart failure. An off-pump surgical approach was considered but deferred in favor of an on-pump approach to ensure a tension-free, precise anastomosis.

**Figure 3.**
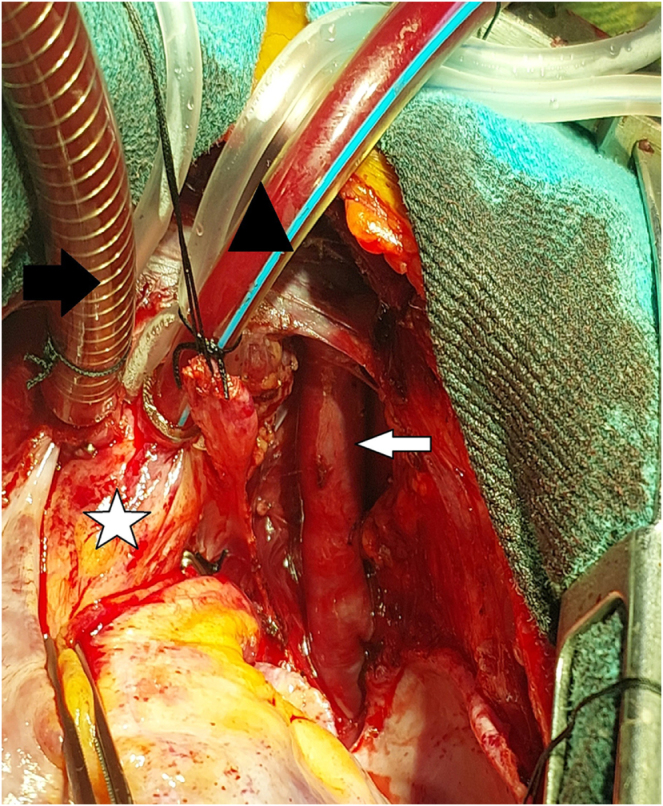
Intraoperative image showing the ascending vertical vein (The white Arrow), the Black Triangle: the arterial cannula, the white star: the ascending aorta, the black arrow: the venous cannula in the superior vena cava.

**Figure 4.**
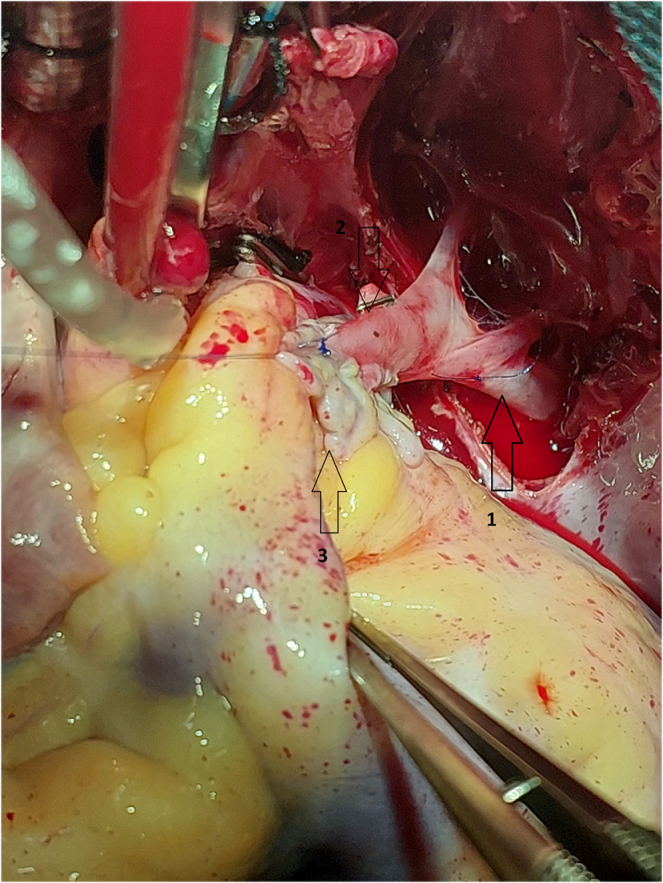
Intraoperative image showing the anastomosis between the ascending vertical vein^[2]^ and the left atrial appendage^[3]^, 1: the left upper pulmonary vein.

The operation was performed by the Cardiothoracic Surgery team through a median sternotomy. Standard cardiopulmonary bypass (CPB) was established with bicaval cannulation. The aorta was cross-clamped, and the heart was arrested using an antegrade cold blood cardioplegic solution. The AVV was carefully isolated and controlled (Fig. 3). The AVV was then transected at its junction with the innominate vein. It was repositioned without kinking and anastomosed end-to-side to a longitudinal incision made in the left atrial appendage using a running 6–0 polypropylene suture (Fig. 4). Patency and lack of tension were confirmed intraoperatively. The remainder of the operation was completed uneventfully, and the patient was weaned from CPB and extubated in the operating room.

The patient’s postoperative course was uneventful. A postoperative TTE showed normal, unobstructed drainage of all four pulmonary veins into the LA, with no detectable gradient across the anastomosis site. She was discharged home on the third postoperative day. At her 1-year follow-up appointment, she was completely asymptomatic, with no exercise intolerance or respiratory infections. A follow-up TTE confirmed sustained normal pulmonary venous flow, a reduction in right heart chamber sizes, and no gradient across the anastomosis.

## Discussion

PAPVC is a rare congenital heart defect with an estimated prevalence of 0.4–0.7%, of which left-sided PAPVC accounts for only 10–15% of cases^[1]^. Patients with isolated left-sided PAPVC, particularly with an intact atrial septum, often remain asymptomatic for years or even decades, leading to frequent delays in diagnosis^[3]^. The diagnostic pathway for this condition has evolved significantly. While cardiac catheterization was once the gold standard, contemporary multi-modality imaging with TTE, CTA, or magnetic resonance imaging (MRI) now provides sufficient anatomical and functional detail for a definitive diagnosis, thereby avoiding the risks of invasive procedures^[6]^. In the present case, CTA was instrumental in precisely delineating the course of the ascending vertical vein and its connection to the innominate vein, confirming the diagnosis non-invasively.

The indications for surgical intervention are well-established in the literature. It is widely accepted that symptomatic patients should undergo repair to prevent the sequelae of chronic right heart volume overload^[1,9]^. Without surgical correction, the persistent left-to-right shunt can lead to progressive right ventricular dilatation and dysfunction, eventual right heart failure, and the development of irreversible pulmonary vascular disease^[1]^. Our patient’s clinical presentation was a clear embodiment of these indications. She was symptomatic with dyspnea, and TTE provided objective evidence of right heart volume overload. This combination of symptoms and clear physiological alteration created a compelling indication for intervention. Given the low perioperative morbidity and mortality associated with surgical repair, proceeding with surgery was the definitive and most appropriate management strategy to avert the natural progression of the disease^[1,3]^.

A critical technical objective of the repair is to create a wide, tension-free, and gradient-free anastomosis between the anomalous pulmonary vein and the left atrium. Postoperative gradients exceeding 4 mm Hg across this anastomosis are associated with significant complications, such as venous thrombosis and stenosis^[4,9,10]^. To optimize conditions for achieving this goal, we elected to perform the anastomosis on cardiopulmonary bypass with cardiac arrest. This approach provided a bloodless, motionless field, allowing for meticulous surgical technique and a precise, tension-free connection, an approach supported by literature demonstrating excellent long-term outcomes^[4,9,11]^. The success of this technical strategy was confirmed at our patient’s 1-year follow-up, where she was asymptomatic, in excellent general condition, and follow-up TTE revealed normal flow without any detectable gradient in the pulmonary veins.

## Conclusion

PAPVC is a rare congenital anomaly that can lead to significant morbidity if left untreated in symptomatic patients. This case illustrates that early diagnosis through a high index of suspicion and advanced non-invasive imaging is key. Prompt surgical intervention in patients with evidence of right heart volume overload prevents the onset of irreversible cardiac complications and ensures excellent long-term outcomes.
